# Revealing climatic and groundwater impacts on the spatiotemporal variations in vegetation coverage in marine sedimentary basins of the North China Plain, China

**DOI:** 10.1038/s41598-024-60838-5

**Published:** 2024-05-02

**Authors:** Yang Liu, Guangdong Wu, Baiheng Ma, Tao Wu, Xinzhou Wang, Qinghua Wu

**Affiliations:** 1 Hebei Key Laboratory of Geological Resources and Environment Monitoring and Protection, Hebei Geo-Environment Monitoring Institute, Shijiazhuang, 050021 China; 2 Changjiang Water Resources Commission of the Ministry of Water Resources of China, Changjiang River Scientific Research Institute, Wuhan, 430010 China; 3 Hubei Key Laboratory of Water Resources & Eco-Environmental Sciences, Wuhan, 430010 China; 4 Hebei Center for Ecological and Environmental Geology Research, Hebei GEO University, Shijiazhuang, 050031 China

**Keywords:** NCP, HB, NDVI, Groundwater depth, Precipitation, Temperature, Ecology, Hydrology

## Abstract

The North China Plain (NCP) is one of the three great plains in China and also serves as a vital region for grain, cotton, and oil production. Under the influence of regional hydrothermal changes, groundwater overexploitation, and seawater intrusion, the vegetation coverage is undergoing continuous alterations. However, a comprehensive assessment of impacts of precipitation, temperature, and groundwater on vegetation in marine sedimentary regions of the NCP is lacking. Heilonggang Basin (HB) is located in the low-lying plain area in the east of NCP, which is part of the NCP. In this study, the HB was chosen as a typical area of interest. We collected a series of data, including the Normalized Difference Vegetation Index (NDVI), precipitation, temperature, groundwater depth, and Total Dissolved Solids (TDS) from 2001 to 2020. Then the spatiotemporal variation in vegetation was analyzed, and the underlying driving mechanisms of vegetation variation were explored in this paper. The results show that NDVI experiences a rapid increase from 2001 to 2004, followed by stable fluctuations from 2004 to 2020. The vegetation in the HB has achieved an overall improvement in the past two decades, with 76% showing improvement, mainly in the central and eastern areas, and 24% exhibiting deterioration in other areas. From 2001 to 2020, NDVI correlates positively with precipitation, whereas its relationship with temperature fluctuates between positive and negative, and is not statistically significant. There is a threshold for the synergistic change of NDVI and groundwater depth. When the groundwater depth is lower than 3.8 m, NDVI increases sharply with groundwater depth. However, beyond this threshold, NDVI tends to stabilize and fluctuate. In the eastern coastal areas, NDVI exhibits a strong positive correlation with groundwater depth, influenced by the surface soil TDS controlled by groundwater depth. In the central regions, a strong negative correlation is observed, where NDVI is primarily impacted by soil moisture under the control of groundwater. In the west and south, a strong positive correlation exists, with NDVI primarily influenced by the intensity of groundwater exploitation. Thus, precipitation and groundwater are the primary driving forces behind the spatiotemporal variability of vegetation in the HB, while in contrast, the influence of temperature is uncertain. This study has elucidated the mechanism of vegetation response, providing a theoretical basis for mitigating adverse factors affecting vegetation growth and formulating rational water usage regulations in the NCP.

## Introduction

Vegetation, acting as a crucial connection between the atmosphere, hydrosphere, and soil, plays a fundamental role within terrestrial ecosystems. It is also an important aspect for indicating global climate change^1^. Moreover, it may provide resources for agriculture, forestry, and livestock, while also offer landscape value for ecotourism and recreational activities. It has important ecological functions, including soil and water conservation, climate and environmental improvement, and biodiversity protection^2,3^. Therefore, vegetation variation is often considered to be an important indicator of ecologically sensitive environments such as water sources, wetlands, forests, grasslands, coastal and estuarine areas.

At present, vegetation change has become an important part in the study of global change, which is of great significance to deeply understand the relationship between vegetation and global change or human activities. To protect ecologically sensitive environments, it is essential to understand what drives vegetation variations^4^. Typically, climate change and human activity are regarded as the leading factors causing the vegetation change^5^.

Climate change plays an important role in the evolution of terrestrial vegetations. Numerous studies have shown that hydrothermal conditions directly influence the regional vegetation growth^6^. In recent decades, the globe has experienced a rapid rise in temperature, and more frequent occurrence of extreme precipitation events^7,8^. Previous literatures have indicated that arise in temperature has a positive influence on the photosynthesis in vegetation^9^, accelerating vegetation growth and improvement. The effect is particularly pronounced at high latitudes^10^. In contrast, at low and middle latitudes, rising temperatures may exacerbate droughts and inhibit vegetation growth^11^. Besides, precipitation plays an important role in influencing inter-annual and intra-annual variations of vegetation. Paruelo and Lauenroth^12^ investigated the relationship between Normalized Difference Vegetation Index (NDVI) and climatic factors in shrublands and grasslands of North American. They highlighted precipitation as a primary controlling factor for NDVI variations in grassland and shrubland ecosystems. Yang et al.^13^ observed a positive relationship between NDVI and cumulative precipitation in spring and summer in North American grasslands, while a negative correlation existed with spring potential evapotranspiration. Except the influence of precipitation and temperature on vegetation variation, there exist to be a certain connection between vegetation and groundwater depth^14^. In cases of ample sunlight and limited precipitation, groundwater is a crucial source of soil moisture, thereby exerting a notable impact on ecosystems. Seeyan et al.^15^ conducted a comparative analysis of vegetation distribution and groundwater levels in the Shaqlawa Basin of northern Kurdistan, Iraq, revealing a significant correlation between groundwater levels and vegetation area at a 95% confidence level. Also, a study conducted by Bhanja et al.^16^ has revealed a significant correlation between NDVI and groundwater levels in natural vegetation-covered areas, including forests, shrubs, and grasslands. However, the connection between NDVI and groundwater levels exhibits great spatial variability based on the geographical location. In regions with high humid, the correlation of vegetation with groundwater levels is predominantly negative, whereas in arid regions, a predominantly positive correlation is observed^17^. Presently, many researchers began to focus on the combined influences of climatic and hydrological factors when studying vegetation changes. Fu and Burgher^18^ demonstrated that the most significant variable influencing NDVI variation is the maximum temperature, followed by precipitation from 28 days prior, and then groundwater levels during the drought period. Calculated NDVI by Naga Rajesh et al.^19^ exhibits significant correlation with surface soil moisture and groundwater, with correlation coefficients of 0.89 and 0.88, respectively. In contrast, a significant negative correlation with surface temperature is found and the correlation coefficient is -0.91.

Vegetation distribution is closely associated with human endeavors, particularly urbanization and farming practices. On the one hand, urbanization and agricultural activities may affect vegetation distribution by altering the water cycle, on the other hand they are the primary catalysts for land conversions^20^, posing a direct influence on vegetation change. In any case the impacts of human activities on vegetation are intricate and varied. The urbanization involves the conversion of a substantial amount of rural land into urban development areas, leading to a reduction in vegetation coverage^21^. At the same time, during the urbanization process, the expansion of green spaces, parks, and urban forests, along with other urban greening facilities, contributes positively to enhancing the urban ecological environment and increasing urban vegetation coverage. As for agricultural irrigation, its impact also has two sides. Agricultural activities typically entail changes in vegetation types and distribution. In general, the increase in irrigation water may enhance the grain production, leading to a rise in the NDVI^22^. However, excessive irrigation may lead to soil salinization and water resource depletion, resulting in land degradation and a decrease in vegetation^20^. For example, in the North China Plain, many rivers’ discharges are smaller than necessary water volume for irrigation, industrial and domestic water use, so over-pumping and drought has caused a dramatic decline of the groundwater level, and an expansion of the area of saline-alkaline land, imposing an adverse impact on the vegetation growth^23^. However, it is noteworthy that since the completion of the South-to-North Water Diversion project in 2014, the groundwater level in the NCP has tended to be stable, and this recovery is projected to continue in the coming decade^24^. This will further facilitate vegetation improvement in heavily irrigated areas in the NCP.

In-situ techniques are the most precise and reliable approaches for assessing vegetation change, but they are laborious and costly^25^. Since 2000s, remote sensing (RS) has emerged as the primary means for observing changes in vegetation over time, offering high spatial and temporal resolution^26^. Vegetation indices are important indicators that reflect vegetation coverage, primary productivity, and other aspects of vegetation ecology. To date, the NDVI vegetation index is widely used across the world. Despite the increasing number of RS-based vegetation studies, most of these studies are concentrated in inland areas, with relatively low human activity intensity, such as the Qaidam Basin and the Ordos Plateau^27^. There is comparatively less research regarding the evolution of vegetation growth conditions in densely populated coastal regions and their relationships with environmental factors. Besides, in attribution analysis of vegetation cover change, in view of the potential influencing factors, many studies are limited to temperature, precipitation, etc. However, due to the difficulty in obtaining groundwater monitoring data with high spatial and temporal resolution, few studies on the impact of groundwater level and total dissolved solids (TDS)on vegetation cover change are found. The Heilonggang Basin (HB) is situated in the core region of the North China Plain (NCP), adjacent to the Bohai Sea. It is an important region for agricultural production, and also one of the most severely affected areas by groundwater overexploitation in China. Due to geographical limitations, the problem of soil salinization in this area is very serious. In recent years, researchers are more concerned about the changes in the ecological vegetation environment of the HB under a series of measures, such as limiting the extraction and pressure of groundwater and ecological replenishment of the South-to-North Water Diversion project. Therefore, analyzing the trend in vegetation variation and the underlying influencing mechanisms, has great guiding significance for managers to make decisions. In this paper, the HB was chosen, and based on 2001–2020 MODIS NDVI data, 20-year trends in vegetation in the basin were analyzed. Moreover, the study on the correlation of NDVI with precipitation, temperature, groundwater, and TDS in groundwater was conducted. Large amount of groundwater level and total TDS data used in this study. This also means that the we adopted a high-density monitoring network to capture more detailed changes in the groundwater system, providing more accurate and reliable results, enhancing the credibility and practicality of the study. Our work may provide a reference for the maintenance of healthy ecosystem, the adaptation of climate change, and rational water resources management.

## Study area

**Figure 1:**
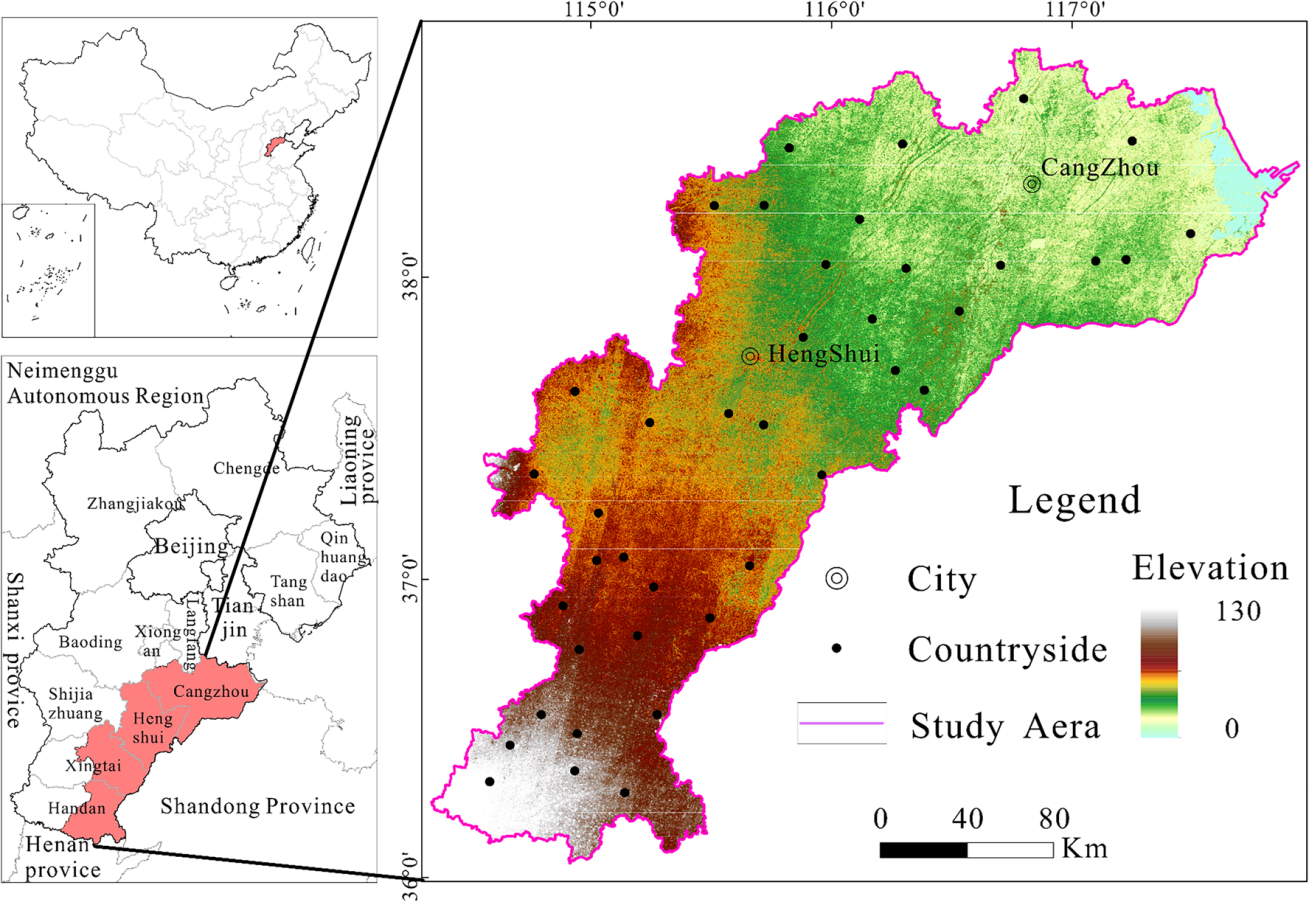
Map of HB location and elevation, created with ArcGIS 10.4 software (www.esri.com).

The HB is situated in the southeastern part of Hebei Province (Fig. 1), encompassing a region of 34,700 square kilometers. It accounts for nearly 1/9 of the NCP. This basin is heavily irrigated, and combinations of increased food demand and declining water availability are creating substantial pressures. In the HB, agriculture accounts for 76% of total water resource consumption, with groundwater supplying over 80% of the irrigation water for agriculture. The primary grain crops are winter wheat and summer maize, while the major cash crops include cotton and peanuts. The region faces a scarcity of water resources, with a per capita water resource availability of only 400–500 m^3^^28^. Given the limited surface water resources, groundwater has emerged as the crucial resource for the economic, social, and agricultural development. The HB has a gentle terrain, gently inclining from the southwest to the northeast, with a topographical gradient ranging from 0.2 to 0.1‰. The landform types from west to east are as follows: piedmont alluvial floodplain, central alluvial lake land, and coastal lowland. Accordingly, the sediment composition transitions from gravel in the western region to moderately coarse sand, medium-to-fine sand in the central area, and fine sand in the coastal zone.

The HB is primarily influenced by a temperate continental monsoon climate, with a yearly mean temperature that spans from 8 to 15 °C, and an annual average rainfall of 500–600 mm. Precipitation is concentrated in July and August, exhibiting warm and precipitation-rich summers, and cold and arid winters. The basin comprises the Zhangwei River system, Heilonggang River system, and Ziya River system, with all rivers ultimately flowing into the Bohai Sea in eastern China. Due to low-lying terrain and inadequate drainage, coupled with the influence of monsoon climate and the geological conditions of low-lying alluvial and coastal plains, it has historically been one of the area most prone to frequent droughts and floods in the NCP and one of the most severely affected regions by salinization.

**Figure 2:**
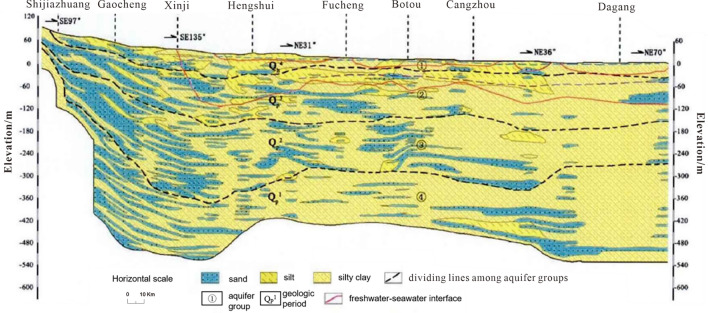
Profile of aquifer groups in the study area^29^.

The aquifer system in the HB is predominantly composed of Quaternary geological formations. The aquifer system of the Quaternary period can be categorized into four distinct aquifer groups (Fig. 2). Of these, the first aquifer group is the primary focus of this study, consisting of loose sediments. The geological composition of the aquifer zone in this region is primarily characterized by the presence of silty sand and silty clay materials. Shallow freshwater is mainly distributed within the HB’s western and northern regions, while shallow brackish water is primarily found in the eastern HB. The distribution of shallow freshwater covers 51.6% of the HB. Precipitation is the primary source of replenishment for both rivers and shallow groundwater.

## Data and method

### Data

#### NDVI

NDVI is currently the most common indicator for describing vegetation change^30^. The NDVI value ranges from -1 to l. As the NDVI increases, it indicates more favorable conditions for vegetation growth, while a decrease in NDVI suggests less favorable conditions for vegetation growth. The NDVI used in this paper is derived from MOD13Q1 remotely sensed data, which is freely available at NASA.com (https://ladsweb.modaps.eosdis.nasa.gov/). The remote sensing data is characterized by a spatial resolution of 250 m. Additionally, it has a temporal resolution of 16 days. A total of 20 periods of the annual average MOD13Q1 remote sensing data from 2001 to 2020, were selected for this paper.

#### Meteorological data

Meteorological data include annual precipitation and temperature from 2001 to 2020. The precipitation data were obtained from Google Earth Engine/UCSB-CHG/CHIPS/DAILY with a resolution of 5 km, and the temperature data were obtained from Google Earth Engine/MODIS/061/MOD11A1 with a resolution of 1 km.

#### Groundwater depth and TDS data

The monitoring data of 192 groundwater monitoring wells in the HB from 2001 to 2020 were collected from the Statistical Yearbook of Groundwater Levels compiled by the China Institute of Geo-Environmental Monitoring (CIGEM). In addition, the groundwater quality data comes from 2020 year, and a total of 46 sets of water samples were collected from the shallow groundwater in the study area and sent to the Hebei Provincial Water Environment Testing Center for testing with national standards (Table 1).

**Table 1:** The list of data sources.

Data	Quantity or accuracy	Unit	Data sources
NDVI	250 m		MOD13Q1 remotely sensed data (https://ladsweb.modaps.eosdis.nasa.gov/)
Precipitation data	5 km	mm	Google Earth Engine /UCSB-CHG/CHIPS/DAILY
Temperature data	1 km	°C	Google Earth Engine /MODIS/061/MOD11A1
Groundwater depth	192wells	m	the China Institute of Geo-Environmental Monitoring (CIGEM)
TDS of groundwater	46wells	g/L	Hebei Provincial Water Environment Testing Center

### Method

#### Maximization processing

The time resolution of MOD13Q1 data is 16d, and there are 2 periods of data per month on average. Before obtaining NDVI, data preprocessing was conducted, including atmospheric correction, radiometric calibration, image registration, and other steps to eliminate noise and bias in the data. Due to the time resolution of NDVI being 16 days, in order to eliminate the effects of clouds, atmosphere and solar altitude angle, the maximum value composites (MVC) method is used to maximize the NDVI data of 2 periods per month, to determine the maximum value of monthly raster data, and to calculate the yearly average value.

#### Geostatistical methods

ArcGIS 10.4 software kriging interpolation was utilized to interpolate the groundwater depth and TDS, respectively. Due to the different spatial resolutions of NDVI and precipitation, temperature, groundwater depth, TDS, etc., before conducting statistical analysis, their spatial resolutions need to be adjusted to be consistent. For example, the spatial resolution of NDVI is 250 m, while the spatial resolution of precipitation is 5 km. The spatial resolution of NDVI needs to be changed to 5 km, keeping the same with precipitation pixels. It is noted that the image element size of the groundwater depth and the vegetation cover distribution map should be consistent.

#### Theil-Sen trend analysis and Mann–Kendall trend test

We adopted a combination of Theil Sen trend analysis and Mann–Kendall significance test. The method can provide more comprehensive and reliable time series trend analysis, and it has good noise resistance, no strict requirements for data continuity. It remains independent of the data distribution pattern., and can effectively avoid the interference of outliers. At present, this approach is widely used in the analysis of vegetation long-time series.

In our study, the Theil-Sen method was employed for the computation of formula:

1 $$\beta =mean\frac{{NDVI}_{j}-{NDVI}_{i}}{j-i}, \forall j>i$$

where: $\beta$-NDVI trend. If $\beta >0$ indicates an increasing trend in NDVI, while $\beta <0$ conversely indicates a decreasing trend. In this study, the $\beta >0.001$ area is classified as an improvement area, the $\beta <-0.001$ area is classified as a degradation area, and the $-0.001<\beta <0.001$ area is classified as a stabilization area; $i<j$ -Time ordinal number; $NDV{I}_{i},NDV{I}_{j}$-NDVI value in the first year; $Mean$-median function.

The Mann–Kendall technique was employed to assess the importance of the pattern of NDVI in the study area with the following process of trend test method: The original hypothesis ${H}_{0}$ is that the temporal data series (${x}_{1},{x}_{2},\dots {x}_{n}$), it is a sample of $n$ independent, random variables with the same distribution, i.e., no notable trend is evident.; Assumption ${H}_{1}$ is a bilateral test series, there is an upward or downward monotonic trend, for all $i,j\le n$ and $i<j$. Define the statistic $S$:

2 $$S={\sum }_{i=1}^{n-1}{\sum }_{j=i+1}^{n}sgn({NDVI}_{i}-{NDVI}_{j})$$

3 $$sgn({NDVI}_{i}-{NDVI}_{j})=\left\{\begin{array}{cc}1,& {NDVI}_{i}-{NDVI}_{j}>0\\ 0,& {NDVI}_{i}-{NDVI}_{j}=0\\ -1,& {NDVI}_{i}-{NDVI}_{j}<0\end{array}\right.$$

where: $S$ -NDVI time series data statistics;$N$ -number of samples; $sgn$-sign function. When $n\ge 10$, the statistic $S$ follows the standard normal distribution closely, the trend test is performed using the test statistic $Z$, which is computed using Eq. (4):

4 $$Z=\left\{\begin{array}{cc}\frac{S}{\sqrt{Var(S)}}& (S>0)\\ 0& (S=0)\\ \frac{S+1}{\sqrt{Var(S)}}& (S<0)\end{array}\right.$$

where: $Z$ -significance statistic;${\text{Var}}({\text{S}})$ -variance function,${\text{Var}}\left({\text{S}}\right)={\text{n}}({\text{n}}-1)(2{\text{n}}+5)/18.$ In a two-sided trend test, considering a specified confidence level $(\mathrm{\alpha })$, if $\left|Z\right|\ge {Z}_{1-\alpha /2}$, the initial hypothesis ${H}_{0}$ is not tenable in such a case, i.e., a noticeable upward or downward pattern can be observed in the time series data for $\mathrm{\alpha }$. A $Z$-value greater than zero signifies a rising trend, whereas a $Z$-value less than zero signifies a declining trend. In this paper, $\mathrm{\alpha }$ is 0.05, i.e., $\left|Z\right|\ge 1.96$ indicates that the significance test has been successfully cleared.

#### Pearson correlation calculation

Correlation analysis is a viable approach to study the relationship between two specific variables. In order to examine the impact of groundwater depth on vegetation cover, this paper takes the image element as the computational unit, and the statistical relationship between vegetation cover and groundwater depth from 2001 to 2020 is obtained by using the raster calculator of ArcGIS10.4. The computational equation is depicted as follows:

5 $$R=\frac{\sum ({x}_{i}-\overline{x })({y}_{i}-\overline{y })}{\sum {({x}_{i}-\overline{x })}^{2}\sum {({y}_{i}-\overline{y })}^{2}}$$

where ${x}_{i}$ is the vegetation cover in 2001 and 2020; ${y}_{i}$ is the depth to groundwater in 2001 and 2020; $\overline{x }$ is the average of the vegetation cover in these 2 years; and $\overline{y }$ is the average of the depth to groundwater in these two years.

## Results

### Spatial and temporal variation of NDVI

**Figure 3:**
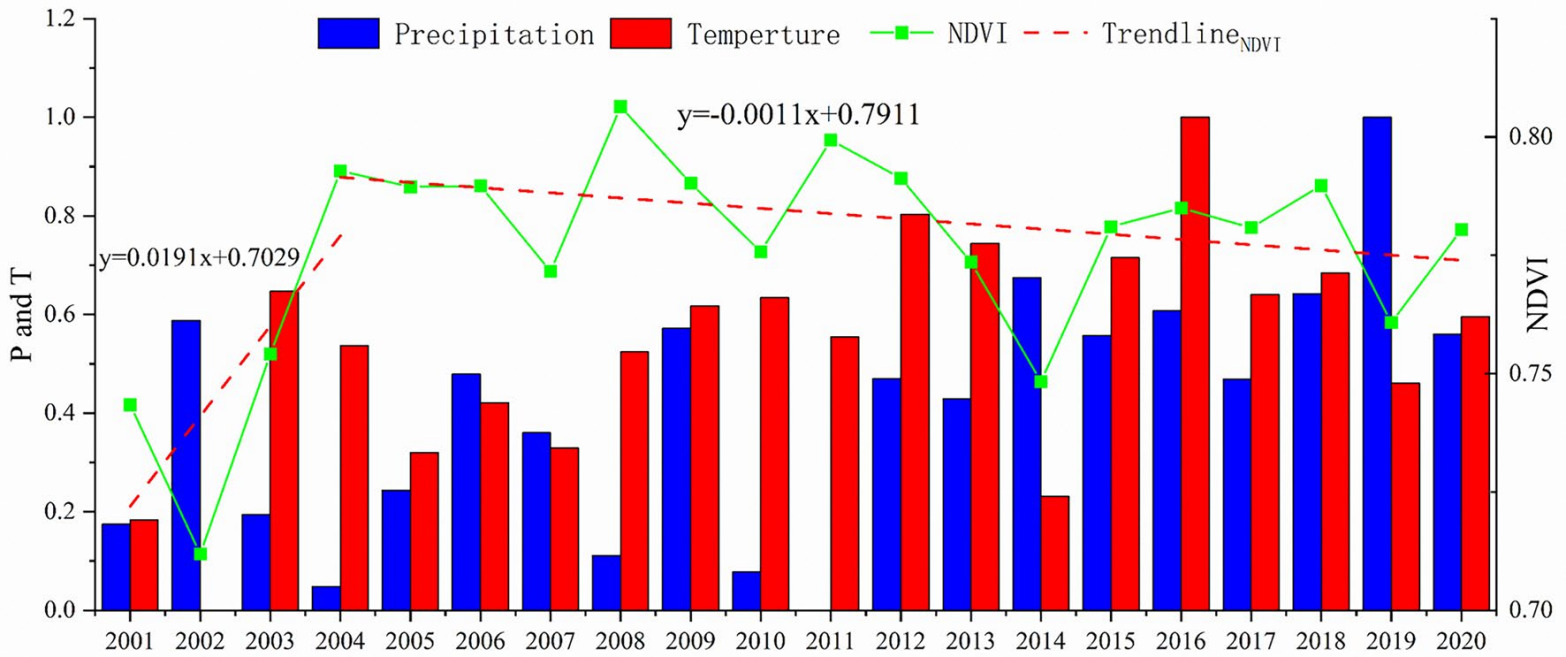
Variation of the NDVI, precipitation and temperature from 2001 to 2020.The temperature and precipitation series have been in normalization.

The annual NDVI in the HB ranges from 0.712 to 0.806 between 2001 and 2020, with an average value of 0.773. The minimum value is 0.712, occurring in 2002, and the maximum value is 0.806, found in 2008.The NDVI variation from 2001 to 2020 can be broadly categorized into two phases: from 2001 to 2004, there was a rapid upward trend in NDVI values, and from 2004 to 2020, NDVI values exhibited minimal fluctuations, remaining in a stable state (Fig. 3).

**Figure 4:**
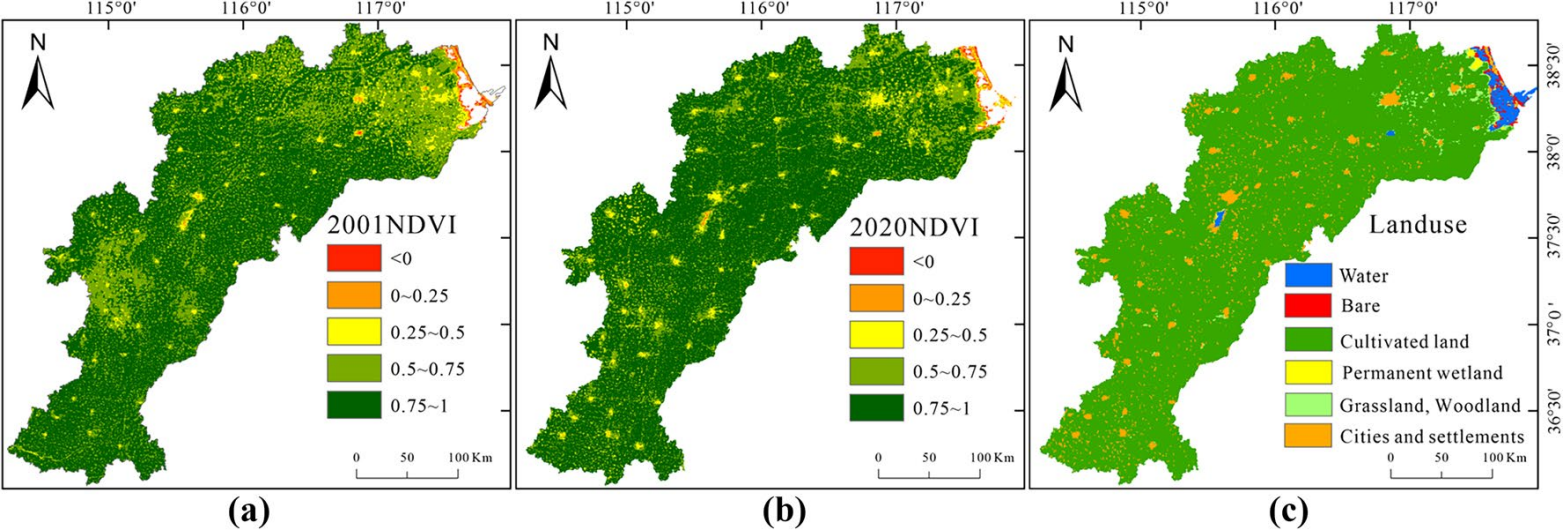
Spatial distribution of NDVI values in 2001 (**a**) and 2020 (**b**), and land use types (**c**) in the HB, created with ArcGIS 10.4 software (www.esri.com).

Spatially, regions with NDVI values below 0.5 are primarily situated in the northeastern of the basin near the Bohai Bay, and sparsely distributed in the urban center areas with human settlements. The region accounts for 4.2% of the whole basin. In contrast, the NDVI in other areas tend to exceed 0.5. In Fig. 4c, the land use distribution is depicted, including six main categories: water bodies, cultivated land, bare land, permanent wetlands, grassland and woodland, and urban and residential areas. Grassland and woodland, along with water bodies, account for 2.9% of the whole basin, mainly found within the eastern coastal regions with a lower NDVI average of 0.52. Urban land covers 6.7% of the area, with an NDVI average of 0.66, surpassing the coastal areas. The remaining majority of the area is cultivated land, constituting over 90% of the whole basin, exhibiting an NDVI average of 0.80.

**Figure 5:**
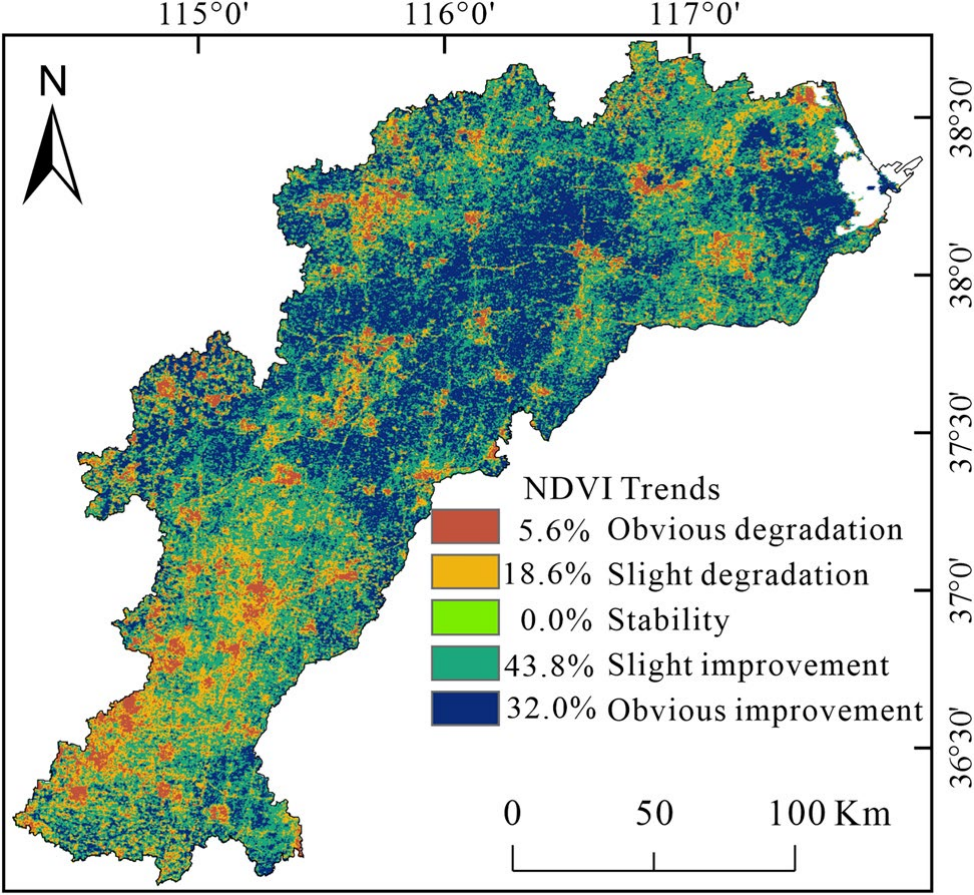
Spatial variation and its area proportion of NDVI trend during 2001–2020 in the HB, created with ArcGIS 10.4 software (www.esri.com).

To study the temporal variation in vegetation, the Theil-Sen trend analysis and Mann–Kendall trend test ware adopted to analyze the NDVI trends during the 2001–2020 period in the HB, and the trends were classified into 5 levels: significant improvement, slight improvement, basic stability, slight degradation, and significant degradation (Table 2). Between 2001 and 2020, the area with increasing NDVI values occupies about 75.8% of the HB, with 32% exhibiting statistical significance. This area is almost concentrated in the central and eastern part of the HB. For example, the NDVI values in the northeastern part of the watershed are generally less than 0.75 in 2001(Fig. 4a), whereas the NDVI values in 2020 are all greater than 0.75(Fig. 4b), indicating a significant vegetation improvement in the coastal zone of the HB. In contrast, the area with decreasing NDVI values represents about 24.2% of the total area, with 5.6% having statistical significance. These decreases are almost distributed in the southern part, followed by urban areas, where urban expansion may result in cropland degradation and its conversion into construction land (Fig. 5).

**Table 2:** Theil-Sen trend analysis and Mann–Kendall trend test Classification Criteria.

Parameter	$\left|{\varvec{Z}}\right|<1.96$	$\left|{\varvec{Z}}\right|>1.96$
$\beta >0.001$	Significant improvement	Slight improvement
$-0.001<\beta <0.001$	Basic stability	Basic stability
$\beta <-0.001$	Significant degradation	Slight degradation

### Correlation of NDVI with precipitation and temperature

**Figure 6:**
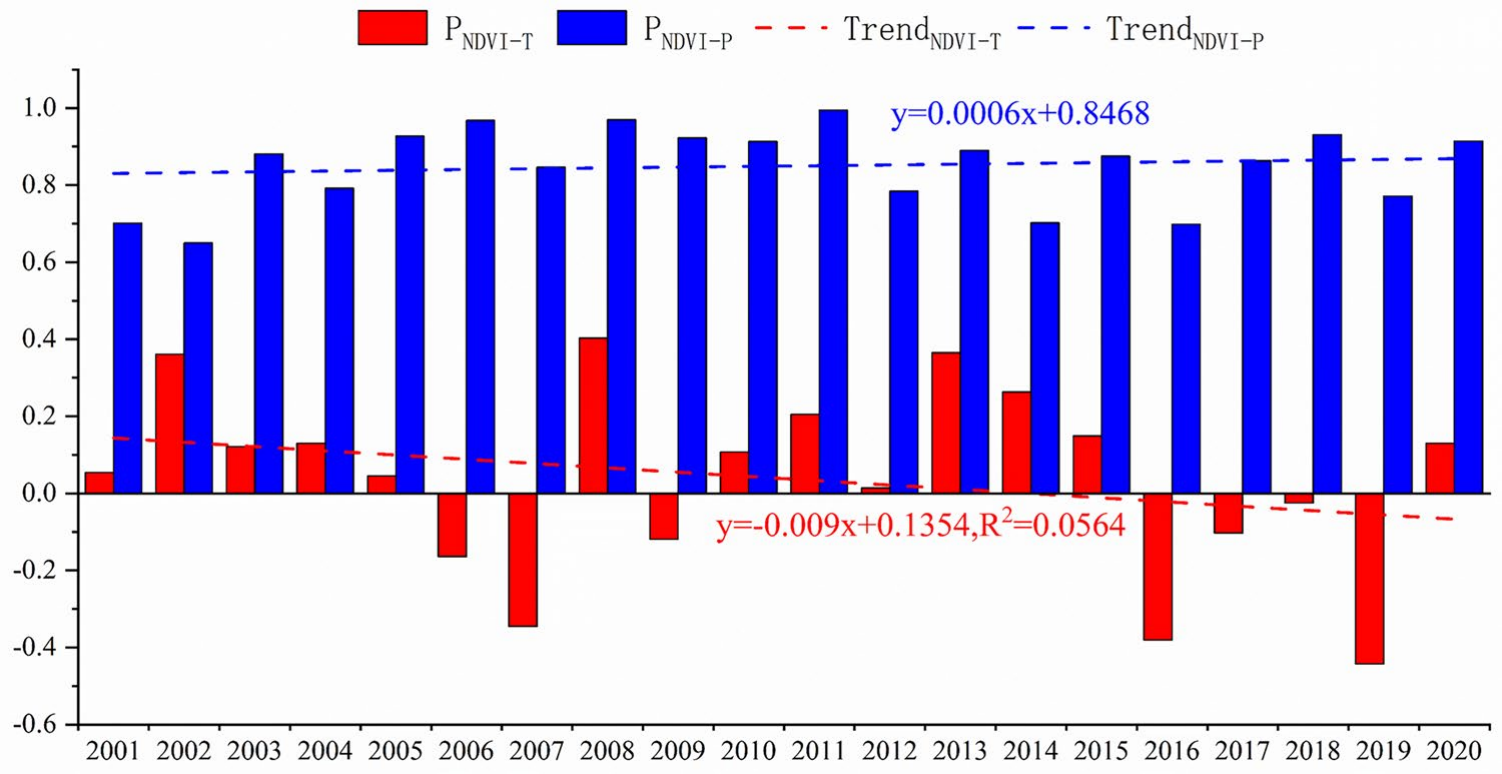
Interannual variations of partial correlation coefficients for NDVI and temperature, represented in red, and for NDVI and precipitation, represented in blue.

The correlations of precipitation and temperature with NDVI have both passed significance tests (P < 0.01), indicating statistical significance. In most years from 2001 to 2020, the partial correlations between NDVI values and precipitation or temperature are positive. This suggests that precipitation and temperature pose a positive influence on vegetation variation during most time. However, the impact of temperature varies significantly with time, and negative effects appear in some years, though they are not statistically significant (Fig. 6).

**Figure 7:**
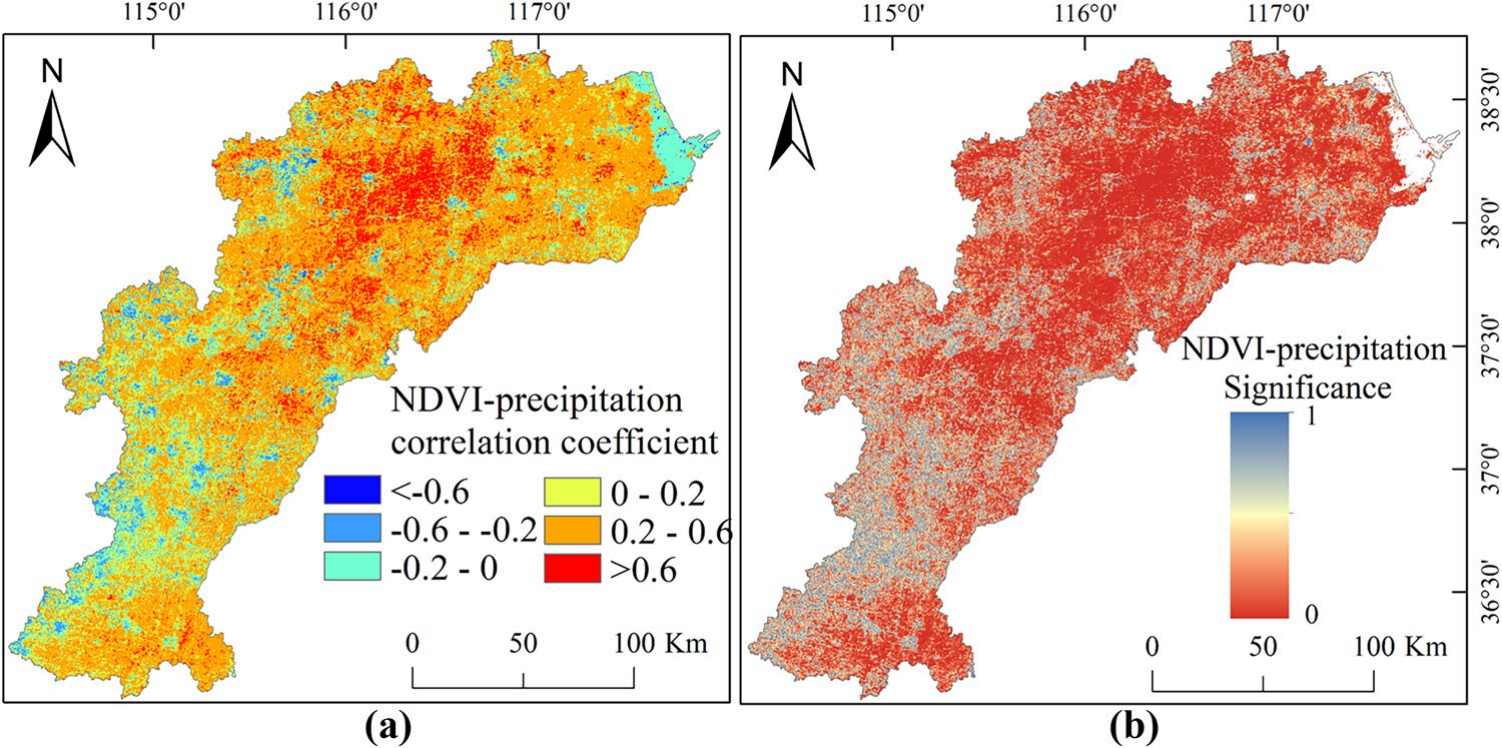
Spatial variability of correlation coefficients (**a**) and significance (**b**) between NDVI and precipitation in the HB, created with ArcGIS 10.4 software (www.esri.com).

**Figure 8:**
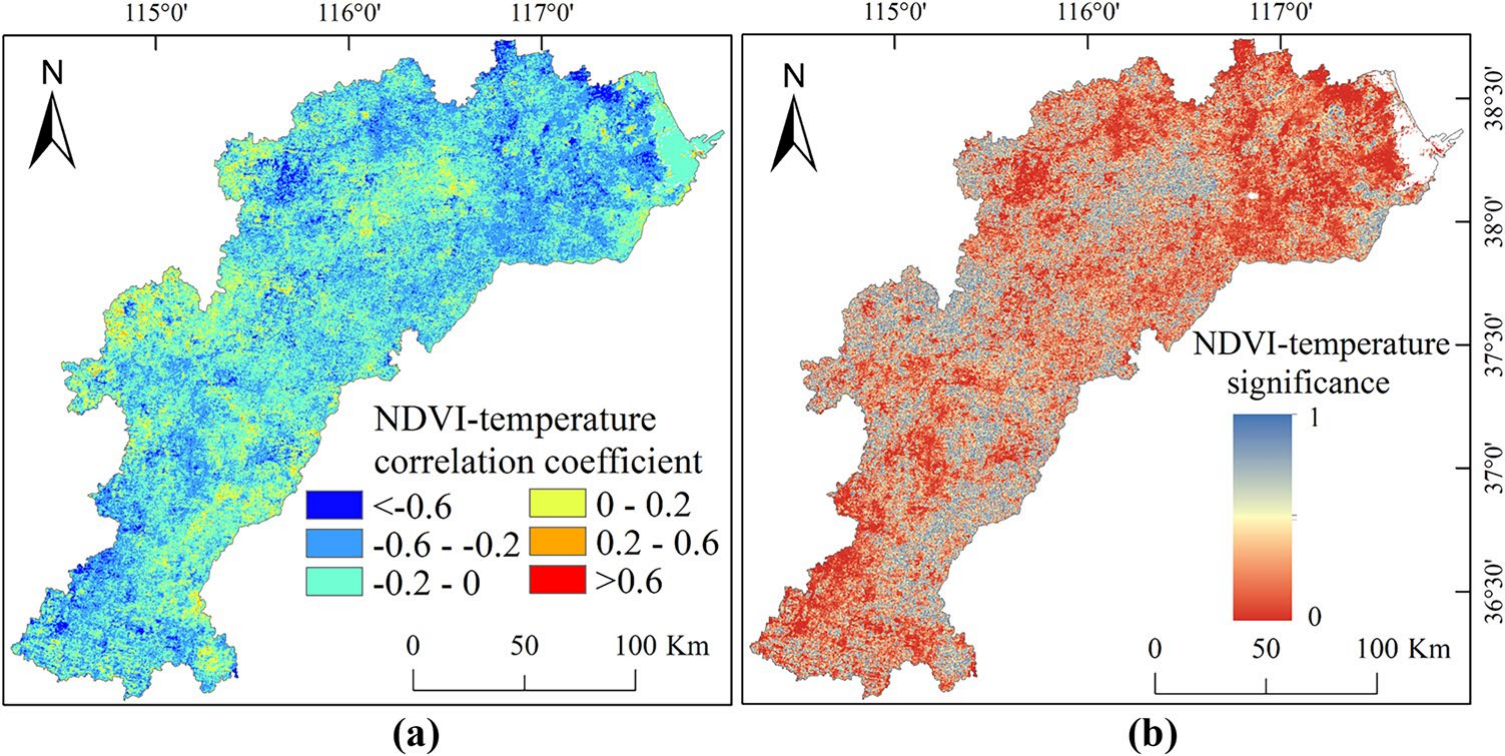
Spatial variability of correlation coefficients (**a**) and significance (**b**) between NDVI and temperature in the HB, created with ArcGIS 10.4 software (www.esri.com).

Partial correlation coefficients for precipitation have an average of 0.854. This signifies an indispensable positive impact of precipitation on vegetation growth. From a spatial perspective, 85.48% of the regions show positive correlations, with 36.31% being statistically significant (*p* < 0.05), primarily in the southern and central-eastern parts of the HB. Spatially, negative correlations for temperature account for 89.15%, with 18.97% being statistically significant (*p* < 0.05), primarily observed within the eastern, northern, and southwestern regions (Figs. 7 and 8).

### Correlation between NDVI and groundwater depth

#### Distribution and changes in groundwater depth

**Figure 9:**
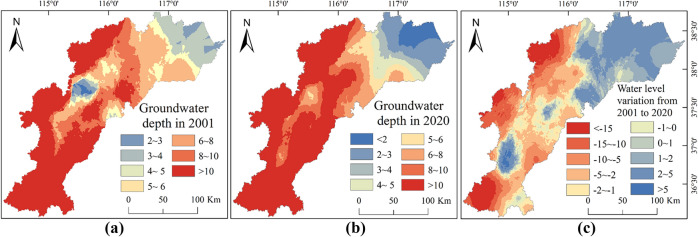
Spatial variability of groundwater depth in 2001 (**a**) and in 2020 (**b**) and groundwater depth variation during 2001–2020 (**c**), created with ArcGIS 10.4 software (www.esri.com).

According to measured groundwater depth data, the eastern coastal areas of the study region exhibit shallow groundwater depths (< 5 m), while in the inland groundwater irrigation areas to the west and south, the groundwater depths become greater (Fig. 9). Spatially, the variation in groundwater depth is predominantly characterized by an increase in depth in the western regions and a decrease in depth in the eastern regions. For example, in the central and eastern areas of the basin in 2001, groundwater depth was relatively shallow, with water table depths less than 4 m. However, by 2020, in the east of the HB, due to the rebound in groundwater levels within the surrounding areas, the distribution area of water table depths less than 4 m expanded further outward. In contrast, in the central area, around Hengshui city, the water table depth decreased, exceeding 4 m. The region exhibiting an increase in groundwater depth covers 60% of the whole basin and is proximately situated in the western, northwestern, and southwestern parts of the HB, with the maximum depth increase exceeding 20 m. The region displaying a decrease in groundwater depth covers 40% and is primarily found within the central and eastern regions, with the maximum depth decrease not exceeding 8 m.

#### Thresholds for the impact of groundwater depth on NVDI

**Figure 10:**
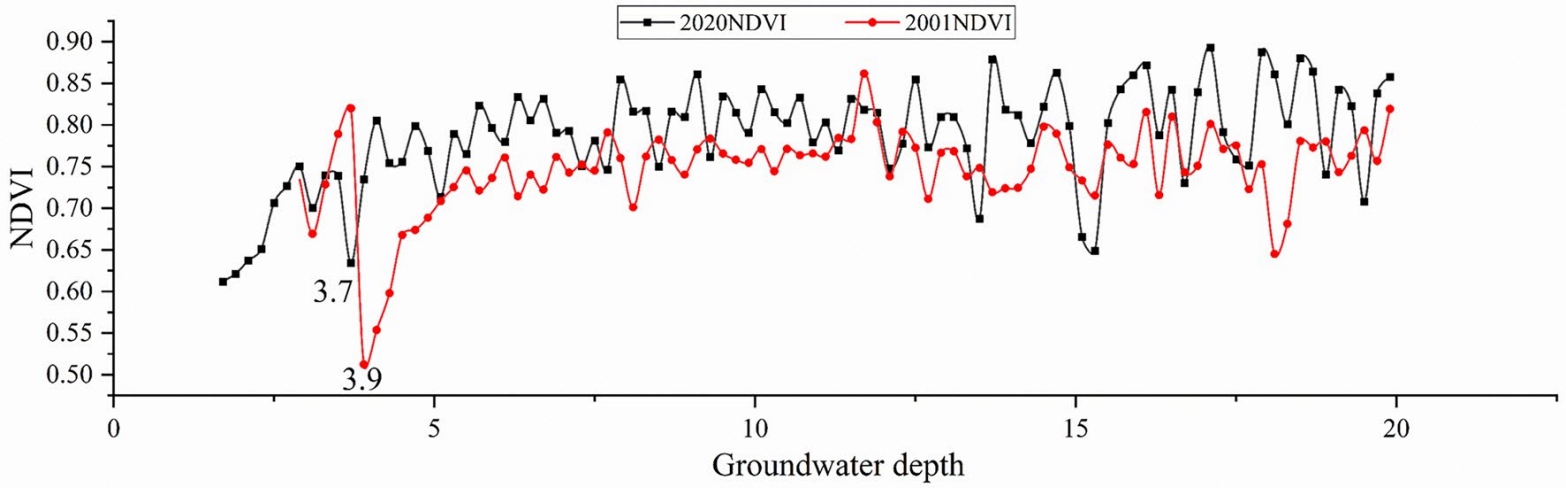
Relationship curve between groundwater depth and mean NDVI values.

The relationship between groundwater depth (at intervals of 0.2 m) and the corresponding NDVI (Fig. 10) reveals that in both 2001 and 2020, the NDVI curves exhibit abrupt changes near a groundwater depth of 3.8 m. When the groundwater depth is less than 3.8 m, the NDVI values are typically below 0.78, and the NDVI values increases rapidly with groundwater depth. As the groundwater depth exceeds 3.8 m, the NDVI values increase to above 0.78 and remain stable thereafter.

#### Spatial analysis of the relationship between NDVI and groundwater depth

The relationship between NDVI values and groundwater depth varies spatially in the HB, indicating differences in the driving mechanisms of vegetation growth in different areas. In the eastern coastal areas of the HB, where groundwater depth is typically lower than 5 m, there are strong positive correlations (0.6 < r < 1) between average NDVI values and groundwater depth, indicating that NDVI increases as groundwater depth increases. In the central area of the HB, NDVI values exhibit strong negative correlations (− 1.0 < r < − 0.6) with groundwater depth. The primary vegetation type in this region is dominated by crops, and NDVI decreases as groundwater depth increases. In the southern and the western parts of the HB, NDVI values exhibit strong positive correlations (0.6 < r < 1) with groundwater depth. Groundwater depth in these areas is significantly greater than 10 m, and NDVI increases as groundwater depth increases.

## Discussion

### Human-induced mechanisms of NDVI variations

In reaction to stimuli triggered by natural and anthropological interference, vegetation undergoes continuously dynamic change^31–33^, and the patterns of change vary significantly from region to region. We identified 2004 as an abrupt point in NDVI in the HB, which is also found in previous literatures. For example, Cao Yanping et al.^34^ found a rapid increase in annual NDVI values from 2001 to 2004, followed by stabilization. This may be associated with a range of government policies in China. One such policy is the “Return of Cropland to Forests” project, which commenced in the late 1990s. This initiative aimed to increase vegetation cover by afforestation^8^. So far, these projects have improved the degraded vegetation and consequently led to a rapid increase in NDVI values.

**Figure 11:**
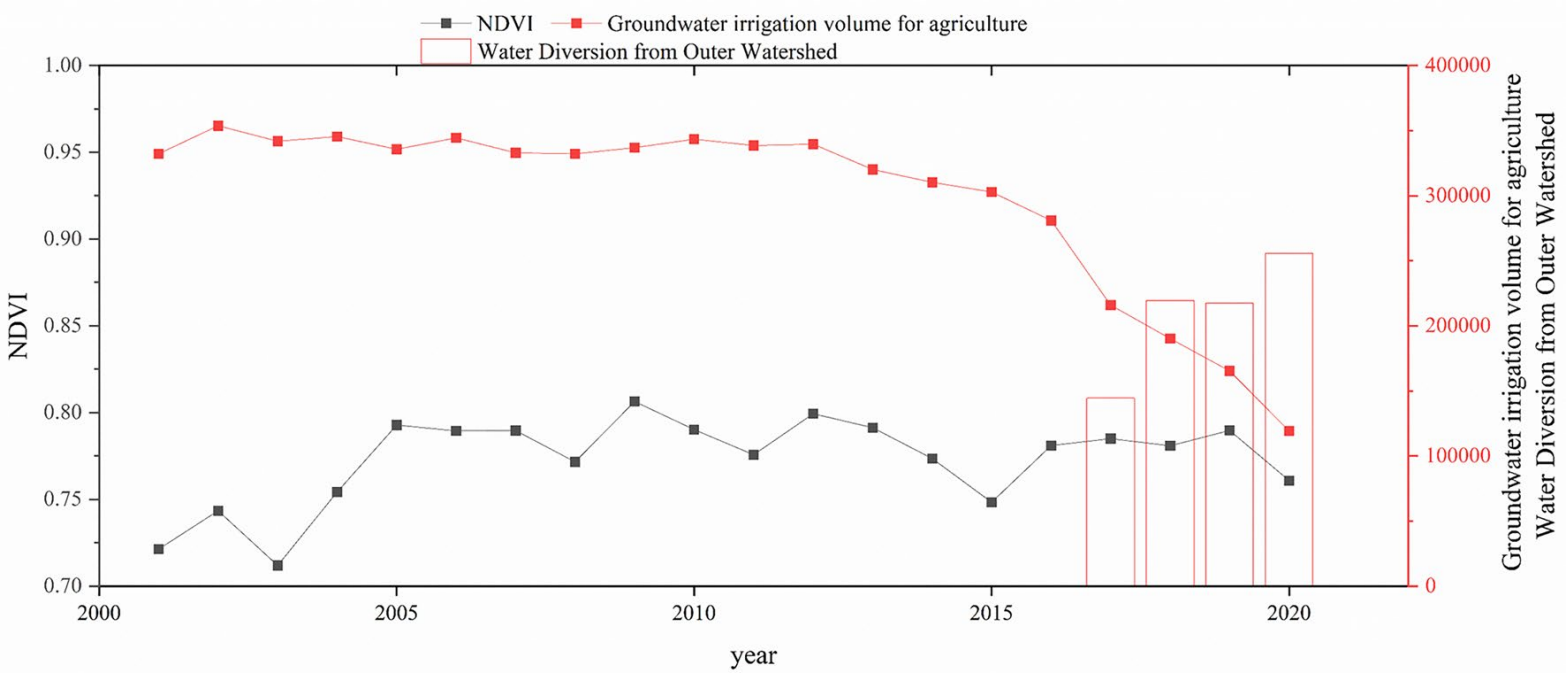
The relationship between groundwater agricultural irrigation and NDVI over 20 years, after the South-to-North Water Diversion Project began operation in 2017, resulted in increased water transfer from the external drainage basins.

Besides, human activities may play a vital role in vegetation alteration^30^. The distinct spatial distribution patterns of NDVI reflect the close relationships between vegetation growth and anthropologic practices such as urbanization, land use variation. Cities’ expansion may threaten the existence of vegetation ecosystems^35^. Sun et al.^36^ found a significant drop in NDVI in Chinese cities over the past three decades, indicating that urbanization has greatly decreased vegetation cover. This is not entirely the case in China. If reasonable planning and layout of urban construction can be carried out, vegetation coverage can even be improved. For example, in human activity areas of the Qilian Mountains, NDVI exhibited a consistent upward trend, signifying a gradual improvement of ecological environment^30^. In addition to the direct transformation of the land surface, human activities may affect the change of terrestrial vegetation by altering the water cycle (mainly the groundwater hydrological processes in the HB). The South-to-North Water Diversion Project increases surface water availability by supplying water to the Fuyang River basin, reducing groundwater usage, and improving the local environment, indirectly benefiting agricultural production^37^. After 2017, with the official replenishment of water from the South-to-North Water Diversion Project to the HB basin, the groundwater extraction rate has continued to decrease, but the Normalized Difference Vegetation Index (NDVI) has remained at a relatively high level (Fig. 11). The widespread use of surface water gradually replacing groundwater extraction will profoundly impact the hydrological cycle patterns in the HB basin and even the NCP in the future. The "Groundwater-induced mechanisms of NDVI variations" is independently separated for discussion and analysis below.

### Climate-induced mechanisms of NDVI variations

Vegetation growth is often influenced by climate change, especially variations in precipitation and temperature^38^, but the extent and mechanisms of their impacts differ significantly. Chuai et al.^39^ found the relationship between NDVI and temperature, as well as precipitation, exhibited distinct differences in Inner Mongolia. In our study, precipitation plays a more substantial role in altering trends in vegetation growth, compared to temperature. This is supported by some studies where, among the factors influencing NDVI in mid-latitude regions, precipitation is the primary factor, while temperature is typically secondary^11,40–42^. However, sometimes precipitation may exert an adverse influence on vegetation growths. For example, in humid regions, a rise in precipitation may contribute to a reduction in both temperature and radiation, thereby inhibiting the growth of vegetation^43^. Yuchen et al.^30^ found that in the Qilian Mountains, temperature stands as the predominant controlling factor for the development of vegetation, and it exerts control over a region with an area proportion of 63.81%, significantly surpassing the area proportion of 29.95% controlled by precipitation.

The impact of temperature on vegetation variation is exceedingly intricate. Rising temperatures facilitates the growth of specific vegetation by supplying the essential heat required for their growth^9^, notably in mountainous or high-latitude areas. Nevertheless, the enhanced evapotranspiration resulting from temperature increase may intensify the drought, thereby restricting vegetation growth^44,45^, especially in arid and semi-arid, or middle-low latitude areas^40^. In general, regions with abundant precipitation exhibit a significant and positive association between vegetation productivity and temperature, whereas in areas where precipitation is insufficient, the negative correlation between NDVI and temperature becomes more pronounced^46^. Thus, temperature rise does not always facilitate plant growth. Moreover, the beneficial impact of climate warming on plant growth may decrease with time^47,48^. This also explains why, in our study, from 2001 to 2020, the correlation of temperature and NDVI is highly spatially and temporally variable. It can be seen that the effects of temperature and precipitation on NDVI need to be analyzed together.

### Groundwater-induced mechanisms of NDVI variations

Similar to meteorological factors, groundwater also plays a crucial supportive role in vegetation growth^49,50^, despite its close relationship with meteorological conditions and human activities^51^. Climate change, land cover change and the over–abstraction of groundwater threaten the existence of groundwater-dependent ecosystems. Water availability is a predominant determinant of vegetation growth^52^. In arid environments, ecosystems with a higher groundwater level (e.g., a spring or groundwater seepage) are more diverse ecologically^35^, being regarded as biodiversity hotspots. But the impacts of different groundwater depths on vegetation growth may differ spatially. Fu and Burgher^18^ conducted a correlation analysis of riparian NDVI and environmental variables, and the results indicated that larger areas with high NDVI occurred when groundwater depth was less than 16 m. In our study, a groundwater depth threshold of 3.8 m was established, indicating that groundwater is unfavorable for surface vegetation growth when the depth is less than 3.8 m. Thus, in order to maintain and enhance vegetation growth, it is advisable to control groundwater depth at a level greater than 3.8 m. This also provides an important reference for the scientific and reasonable implementation of ecological water replenishment of the South-to-North Water diversion project.

**Figure 12:**
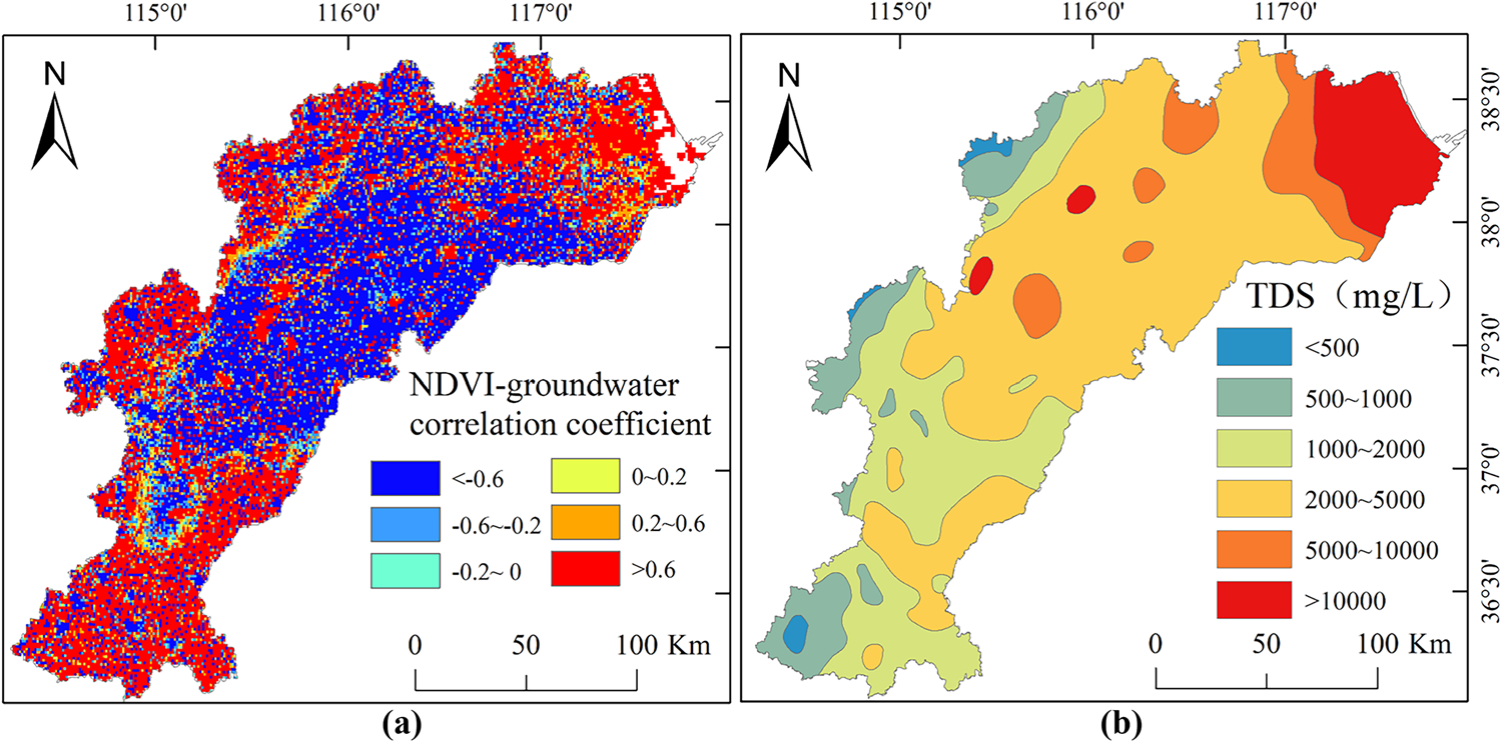
Spatial distribution of the (**a**) correlation coefficient between groundwater depth and NDVI value, (**b**) dissolved total solids (TDS) concentration in groundwater, created with ArcGIS 10.4 software (www.esri.com).

Sommer and Froend^51^ argued that groundwater depth serves as a surrogate for precipitation, soil moisture, and human replenishment or extraction, making it the most critical abiotic factor driving vegetation changes in southwestern Western Australia. Our study results provide supporting evidence for this perspective as well. Nevertheless, within the entire study region, the relationship between groundwater depth and NDVI exhibits pronounced regional variability (Fig. 12).

In the eastern coastal region of the HB, groundwater depth is typically less than 5 m. Considering the evaporation limit of 5 m, evaporation decreases as groundwater depth increases. Intense evapotranspiration processes may lead to rapid moisture depletion in this area, while salts from groundwater remain in the shallow soil, causing soil salinization and even soil compaction. These factors contribute to inferior vegetation growth with shallower groundwater depths. This explains why the NDVI in the eastern coastal region is almost the smallest. The phenomenon in the HB contrasts significantly with arid regions or desertified areas in Northwestern China, where the predominant vegetation consists of salt-tolerant plants like shrubs, and water availability is the determinant influencing vegetation growth. Groundwater exerts a positive influence on terrestrial vegetation growth in these regions^53^. Although coastal areas are also the primary distribution zones for wetland vegetation or salt-tolerant wetland plants, there are unique factors at play. On one hand, these types of vegetation are less sensitive to water availability. On the other hand, exceptionally high TDS levels, exceeding 10 g/L at their maximum, due to seawater intrusion, have a negative impact on vegetation growth. Saline groundwater (TDS > 2 g/L) hinders vegetation growth, and as groundwater depth decreases, groundwater TDS increases, leading to lower NDVI values. However, the coastal vegetation has improved significantly in the past two decades, which may also be related to the implementation of the South-to-North Water Diversion project. Since the South-to-North Water Diversion Project was fully completed in 2014, water from the South has become the main water source for many cities in the HB. The project has greatly alleviated the contradiction between supply and demand of water resources by implementing ecological water replenishment for rivers along the line and reducing groundwater over-exploitation in the HB. The increase in water levels in many rivers and the recovery of groundwater level have effectively curbed the further intrusion of seawater, and groundwater quality has been significantly improved, promoting the recovery of vegetation.

In HB’s central part, there is a strong negative relationship between NDVI values and groundwater depth. Unlike the coastal areas, groundwater depth in this region is generally greater than 5 m. Shallow groundwater exhibits slightly lower TDS levels (0.5 < TDS < 2 g/L), with predominance of shallow freshwater. However, freshwater resources are limited, and saline water is mainly distributed below shallow freshwater and above deep freshwater, typically at depths ranging from 50 to 160 m. There is no large-scale abstraction of groundwater for irrigation in this area. Typically, vegetation growth is associated with soil moisture conditions, and a positive correlation exists between surface soil wetting or drying and the greening or degradation of vegetation^54^. Vegetation growth in this region is influenced by groundwater, as plant roots absorb groundwater through capillary action. As groundwater depth increases, the availability of freshwater resources to vegetation decreases, resulting in poorer growth conditions. Therefore, increasing groundwater depth leads to reduced soil moisture, and consequently, poorer vegetation growth.

In the southern and western parts of the HB, NDVI values exhibit a positive correlation with groundwater depth. Considering that freshwater resources in this area (TDS < 1 g/L) are relatively abundant, but groundwater extraction is intense, with the extracted groundwater primarily used for agricultural irrigation, thereby promoting the positive growth of surface vegetation. Intensive groundwater irrigation leads to a decline in the groundwater table; nevertheless, irrigated crops generally display elevated NDVI values compared to natural vegetation or rain-fed crops^55^, resulting in larger NDVI values in areas with higher extraction rates and increasing groundwater depth. Besides, due to the large groundwater depth of more than 10 m in the southern and western HB, the implementation of the South-to-North Water Diversion project and the groundwater extraction restriction policy can only slow down the downward trend of groundwater, or curb the further decline of local groundwater level. In the past two decades, the implementation of these measures has reduced the use of agricultural water, leading to a decline in agricultural production and the corresponding NDVI.

## Conclusions and prospect

### Conclusions

In this study, a variety of methods, including geo-statistics, linear regression, Theil-Sen trend analysis, Mann–Kendall trend test and correlation analysis were employed. Based on 20-year data series from 2001 to 2020, including MOD13Q1 NDVI vegetation index, precipitation, temperature, groundwater depth, and TDS, our study analyzed the spatiotemporal variations in vegetation growth in the HB and explored their driving mechanisms. The following conclusions are given:

Firstly, the NDVI values vary from 0.712 to 0.806, with an average of 0.773. From 2001 to 2004, it exhibited a rapid increasing trend, while after 2004, the fluctuation stabilized. The vegetation shows an overall improvement, with 76% of the vegetation exhibiting an improving trend, mainly in the central and eastern parts, while 24% of the vegetation showed degradations, primarily in the southern and urban areas. There is significant regional variation in groundwater depth changes. The region experiencing a growth in groundwater depth constitute for 60% and is predominantly situated in the western, northwestern, and southwestern parts of the HB, with maximum changes exceeding 20 m. The area with decreasing groundwater depth accounts for 40% and is mainly distributed in the central and eastern parts, with the maximum depth reduction not exceeding 8 m.

Secondly, the NDVI values from 2001 to 2020 are significantly positively correlated with precipitation, but the relationship between NDVI and temperature varies greatly in different years; in some years, it is positively correlated, while in others, it is negatively correlated, and the correlation is not significant. It is speculated that the influence of temperature on NDVI may be disturbed by other factors such as precipitation. Moreover, there is significant spatial variability in the correlation between NDVI values and precipitation and temperature. The critical point of NDVI co-variation with groundwater is 3.8 m, with NDVI averaging an increase with increasing groundwater depth, but stabilizing and fluctuating after exceeding 3.8 m.

Thirdly, in the eastern coastal area of the HB, the groundwater depth is less than 5 m, and the average NDVI is strongly positively correlated with groundwater depth. The variation in NDVI is primarily influenced by the surface soil TDS controlled by groundwater depth. In the central area, where groundwater depth ranges from 5 to 10 m, they exhibit a strong negative correlation (− 1.0 < r < -0.6). NDVI variations are mainly influenced by soil moisture conditions controlled by groundwater depth. In the western and southern parts, where groundwater depth exceeds 10 m, they exhibit a strong positive correlation (0.6 < r < 1). NDVI variations are mainly influenced by the intensity of agricultural irrigation.

### Prospect

The HB is heavily irrigated, having a strong demand for irrigation. However, the amount of surface water in this region is far from sufficient to meet the needs of vegetation growth, necessitating reliance on groundwater to sustain vegetation growth. Groundwater is a vital water source for vegetation growth, and during the dry season, it can provide the necessary water for plants. Excessive groundwater extraction or the excessive discharge of pollutants can also lead to a decline in groundwater quality, which may have adverse effects on vegetation. During the past two decades, the implementation of the South-to-North Water Diversion project and a series of ecological restoration policies have already altered groundwater dynamics, thereby posing an important influence on vegetation coverage in the HB. But rising groundwater levels are not always good things for terrestrial ecosystem health. Therefore, keeping the groundwater depth within a reasonable range is essential for maintaining the health of vegetation and the balance of ecosystems. In the future, it is necessary to develop a comprehensive framework covering the South-North Water diversion project and ecological restoration policies to draw up measures related to the rational use and sustainable management of groundwater.

In addition, in order to predict the future water cycle, and its associated changes in NDVI, the construction of an eco-hydrology numerical model incorporating both natural and artificial processes is necessary. Moreover, based on this model, the optimal regulation of groundwater resources under different scenarios can provide important guidance for managers in the ecological water replenishment of South-to-North Water Diversion project, and formulation and implementation of ecological restoration strategies.

## Data Availability

The datasets used and/or analyzed during the current study are available from the corresponding author on reasonable request.
